# No Association Between Hamstrings-to-Quadriceps Strength Ratio and Second ACL Injuries After Accounting for Prognostic Factors: A Cohort Study of 574 Patients After ACL-Reconstruction

**DOI:** 10.1186/s40798-023-00670-9

**Published:** 2024-01-12

**Authors:** Johan Högberg, Ramana Piussi, Mathias Wernbom, Francesco Della Villa, Rebecca Simonsson, Kristian Samuelsson, Roland Thomeé, Eric Hamrin Hamrin Senorski

**Affiliations:** 1 Sportrehab Sports Medicine Clinic, Stampgatan 14, 411 01 Gothenburg, Sweden; 2 Sahlgrenska Sports Medicine Center, Gothenburg, Sweden; 3 Unit of Physiotherapy, Department of Health and Rehabilitation, Institute of Neuroscience and Physiology, Sahlgrenska Academy, University of Gothenburg, Box 455, 405 30 Gothenburg, Sweden; 4 Department of Orthopaedics, Institute of Clinical Sciences, Sahlgrenska Academy, University of Gothenburg, Gothenburg, Sweden; 5 The Rydberg Laboratory for Applied Sciences, Halmstad University, Box 823, 301 18 Halmstad, Sweden; 6 Education and Research Department, Isokinetic Medical Group, FIFA Medical Centre of Excellence, Bologna, Italy; 7 Swedish Olympic Committee, Stockholm, Sweden

**Keywords:** Anterior cruciate ligament, Knee injury, Muscle strength, Hamstrings/quadriceps ratio

## Abstract

**Background:**

The stress on the anterior cruciate ligament (ACL) induced by the quadriceps can be attenuated by activation of the hamstrings by exerting an opposing torque to the anterior translation of tibia. Consequently, considering the ratio between strength of the hamstrings-to-quadriceps (HQ-ratio) may be of value to reduce the odds of second ACL injuries. The objective was therefore to evaluate (1) the association between HQ-ratio and the occurrence of a second ACL injury in patients after ACL-reconstruction within 2 years of return to preinjury sport level and (2) to compare the HQ-ratio between males and females after ACL reconstruction.

**Methods:**

Patients who had undergone primary ACL reconstruction and participated in knee-strenuous activity preinjury were included. Demographics, the occurrence of a second ACL injury, and muscle strength test results before returning to preinjury sport level were extracted from a rehabilitation registry. The endpoint was set at a second ACL injury or 2 years after return to preinjury sport level. A multivariable logistic regression was used to analyze the association between the HQ-ratio and a second ACL injury.

**Results:**

A total of 574 patients (50.0% female) with a mean age of 24.0 ± 9.4 years at primary ACL reconstruction were included. In the univariable logistic regression analysis, the odds of sustaining a second ACL injury decreased by 3% for every 1% increase in the HQ-ratio (OR 0.97 [95% CI 0.95–1.00], *p* = 0.025). After adjusting for the time from reconstruction to return to preinjury sport level, sex, preinjury sport level, graft choice, age, and body mass index, the results were no longer significant (OR 0.98 [95% CI 0.95–1.01], *p* = 0.16). Females had a higher HQ-ratio compared with males for both the ACL-reconstructed and uninjured side (3.7% [95% CI 5.7; 1.8%], *p* = 0.0002 and 3.3% [95% CI 4.6; 2.1], *p* < 0.001, respectively).

**Conclusion:**

The HQ-ratio did not significantly affect the odds for sustaining a second ACL injury upon return to preinjury sports level after primary ACL reconstruction. Females had a significant higher HQ-ratio than males for both the ACL reconstructed and uninjured side.

## Key Points

- The HQ-ratio does not significantly affect the odds for a second ACL injury upon return to preinjury sport level.
- The HQ-ratio does not appear to have to be considered in the RTS decision-making.
- Females have a significantly higher HQ-ratio than males for both the ACL reconstructed and uninjured side.

## Background

After an anterior cruciate ligament (ACL) rupture with subsequent reconstruction, achieving symmetrical quadriceps and hamstrings strength [≥ 90% of the uninvolved limb, i.e. limb symmetry index (LSI)] after the ACL reconstruction are considered among the key measures of successful outcomes [1]. However, the LSI may overestimate strength recovery as the achievement of symmetrical muscle strength may in part be due to a decreased absolute strength of the uninjured leg [2, 3]. In addition to assessing the strength of the quadriceps and hamstrings in isolation, to consider their contributions to ACL loading may add an important piece of the ACL rehabilitation puzzle.

During a quadriceps contraction, an anterior tibial translation force is created, which potentially increases the strain on the ACL [4, 5]. The stress on the ACL induced by the quadriceps can be attenuated by activation of the hamstrings, which acts as a synergist to the ACL by exerting an opposing torque to the anterior translation of the tibia [4, 5]. Thus, in addition to the focus on restoring symmetrical knee-joint muscle strength and regaining preoperative strength values, respectively, the consideration of the ratio between the strength of the hamstrings and the quadriceps (the HQ-ratio) may be of value to further reduce the risk of a second ACL injury. In support of this notion, Kyritsis et al. [6] reported significantly lower peak torque values for the hamstrings, but not the quadriceps, in the ACL reconstructed leg in patients who subsequently sustained an ACL graft rupture compared to those who did not. In addition, the same researchers reported that for every 10% decrease in the HQ-ratio, the risk of ACL graft rupture increased by 10.6 times at the time of return to sport (RTS) [6]. This finding is in line with those of Myer et al. [7], who reported a lower HQ-ratio in female athletes who subsequently suffered a first-time ACL injury compared with female athletes who did not go on to suffer ACL injuries. Interestingly, Myer et al. [7] also observed strength differences between males and females, which is further supported by the results of a systematic review, in which the HQ-ratio of was 51.9% ± 8.0% for females and 60.7% ± 9.5% for males when averaged over all angular velocities [8]. However, data with regard to HQ-ratio differences between sexes needs to be complemented as only 11 of the 22 studies included females, with generally small sample sizes for studies which included females [8].

The data that supports a possible association between a low HQ-ratio and an increased risk of a secondary ACL injury is scarce. Furthermore, it is worth noting that the value of the HQ-ratio to predict ACL injuries is currently unclear, as inconsistent findings between multiple studies were observed in a recent systematic review on the relationship between HQ-ratio and the risk of ACL injury [9]. Consequently, due to the very limited data with regard to the possible association between the HQ-ratio ratio and second ACL injuries, the results from Kyritsis et al. [6] warrant replication in order to justify the use of the HQ-ratio as a part of the decision-making prior to RTS. In addition, clarification of potential sex differences in HQ-ratio is also needed.

The aim of this study was to evaluate the association between the HQ-ratio and the occurrence of a second ACL injury in patients after ACL reconstruction within 2 years of returning to preinjury sport level. A second aim was to compare the HQ-ratio between females and males after ACL reconstruction.

## Method

The study was reported in accordance with the REporting of studies Conducted using Observational Routinely-collected health Data (RECORD) Statement [10]. The study was designed as a cohort study based on data from Project ACL, a rehabilitation outcome registry, specific to patients with ACL injury. Participation in Project ACL is voluntary and open to all patients with ACL injury, regardless of treatment choice. Withdrawal from the registry can be made at any time without consequences. Informed consent is obtained, and the rights of participants are protected. The study was carried out in accordance with the Code of Ethics of the World Medical Association (Declaration of Helsinki). Ethical approval has been obtained from the Swedish Ethical Review Authority with registration number 2020-02501.

### Project ACL

More than 4000 patients have enrolled in Project ACL since its inception in 2014. The aim of Project ACL is to improve care for patients with ACL injury by regularly assessing muscle function and patient-reported outcomes (PROs). Patients are assessed according to a predefined schedule starting with ACL injury or reconstruction as a baseline and then at 10 weeks, 4, 8, 12, 18, 24 and 60 months and then every 5 years. At the assessment, patients complete a test battery consisting of isokinetic concentric muscle strength testing in a Biodex System 4 [11], hop performance [12] and PROs [13]. The tests are supervised by physical therapists educated by Project ACL to perform the standardized tests. Data from the tests of muscle function are entered in the Project ACL database by the test leader, while the PROs are recorded by the patients online on the Project ACL website. Demographic information is registered on the Project ACL website upon registration by the patients.

### Patients

Patients eligible for inclusion in the present study were (1) registered in Project ACL after a primary ACL rupture, (2) treated with ACL-reconstruction, (3) participated in knee-strenuous sport [14], i.e. they had a preinjury Tegner Activity Scale (Tegner) score of ≥ 6, (4) reconstructed with a hamstring tendon autograft or a patellar tendon autograft, and (5) evaluated with the quadriceps and hamstrings muscle strength test in the Biodex at the follow-up where the patients had reported that they had returned to preinjury sport level (i.e., rated ≥ 6 on the Tegner scale). Patients who had obtained a score of ≥ 6 on the Tegner scale at 10 weeks after ACL reconstruction were excluded, as it was not deemed possible to safely perform high-level knee-demanding activities at that time point.

The Tegner scale aims to assess the level of knee-strenuous activity, ranging from 1 to 10, where higher values indicate more knee-demanding activities [14]. Return to preinjury sport level was defined as obtaining the same score or higher on the Tegner scale as preinjury.

### Data Collection

Information regarding patient demographics, the occurrence of a second ACL injury and the muscle strength test results closest in time to return to preinjury sport level were extracted from the Project ACL database for further analysis on 23 November 2022.

Isokinetic concentric knee extension and flexion strength measurements were performed in a Biodex dynamometer (System 4, Biodex Medical Systems, Shirley, New York, USA) [11]. After a warm-up and familiarization, a total of 3–4 reciprocal repetitions of concentric knee extension and concentric knee flexion contractions were performed with maximum effort, with 40 seconds (s) of rest between each attempt. For each repetition, a concentric knee extension was immediately followed by a concentric knee flexion. All torque values were corrected for the weight of the patients’ leg and gravity. The test was performed between 0° to 90° of knee flexion, at an angular speed of 90°/s . The peak torque expressed in Newton meters (N m) of knee extension and flexion was used for analysis.

At each follow-up in Project ACL, patients receive a question whether they have sustained a second ACL injury or not since their last follow-up. Second ACL injuries were confirmed by clinical assessment and/or magnetic resonance imaging and added to the database of Project ACL by the responsible physician, physical therapist, or the patients themselves.

### Outcomes

The HQ-ratio was determined by dividing the peak hamstrings torque by the peak quadriceps torque and then multiplying by 100 to express the result as a percentage [15]. The primary outcome of this study was the association between the HQ-ratio on the reconstructed knee and the occurrence of a second ACL injury after returning to preinjury sport level. Nevertheless, the HQ-ratio in itself does not provide information whether the strength in the hamstrings and quadriceps, respectively, is sufficient. Consequently, the peak torque was normalized to body mass and expressed as relative peak torque of the quadriceps and hamstrings (N m kg^−1^) for sub-analyses performed on the patients’ ACL reconstructed leg, as well as the LSI as covariates of the association between the HQ-ratio and second ACL injuries. In addition, sub-analyses were performed on the association between the HQ-ratio on the ACL reconstructed leg for ipsilateral injuries (ACL re-rupture) and the HQ-ratio on the uninjured leg for contralateral injuries. Obtaining the same score or higher on Tegner as preinjury was used as a starting point, with the endpoint set at follow-up 2 years after return to preinjury sport level, unless a second ACL injury occurred.

The secondary outcome was the difference in the HQ-ratio between sexes.

### Statistical Method

Statistical analyses were performed with the SAS Statistics for Windows (Version 9.4, SAS Institute, Cary, NC, USA). Continuous variables were presented with the mean, standard deviations and 95% confidence intervals (CI). Categorical variables were presented with the count and percentages. Alpha level of 0.05 was used. For comparison between categorical variables, Fisher’s exact test was used. Logistic regression analysis was used to investigate the association between the occurrence of a second ACL injury and the HQ-ratio. The HQ-ratio was analyzed as a continuous variable and illustrated by intervals of the outcome based on groups consisting of a similar number of patients. The following prognostic factors for a second ACL injury were accounted for using multivariable analysis: the time from reconstruction to return to preinjury sport level, sex, graft choice, age, Tegner preinjury level and body mass index [16]. In addition, the relative peak torque of the hamstrings and quadriceps of the ACL reconstructed leg, as well as the LSI of the quadriceps and the hamstrings, were analyzed in a post-hoc analysis to investigate whether the relative peak torque or LSI values were associated with the odds of a second ACL injury. For the analysis of ipsilateral injuries, contralateral injuries were excluded, while ipsilateral injuries were excluded when analyzing contralateral injuries. The results of the logistic regression models were presented with odds ratios (ORs), 95% CIs and *p* values. Odds ratios were expressed for every 1-unit increase in the predictor variable. A sensitivity analysis was performed with a 10-unit increase to assess the robustness of our results. To analyze the discriminatory ability of the HQ-ratio value between patients who sustain a second ACL injury and those who do not, as well as comparing the discriminatory ability between the associated factors, a receiver operating characteristic (ROC) curve was produced. The area under the ROC-curve was used to interpret the accuracy with the following reference values: 0.5 = no discrimination, 0.7–0.8 = acceptable, 0.8–0.9 = excellent and > 0.9 = outstanding [17].

## Results

**Fig. 1:**
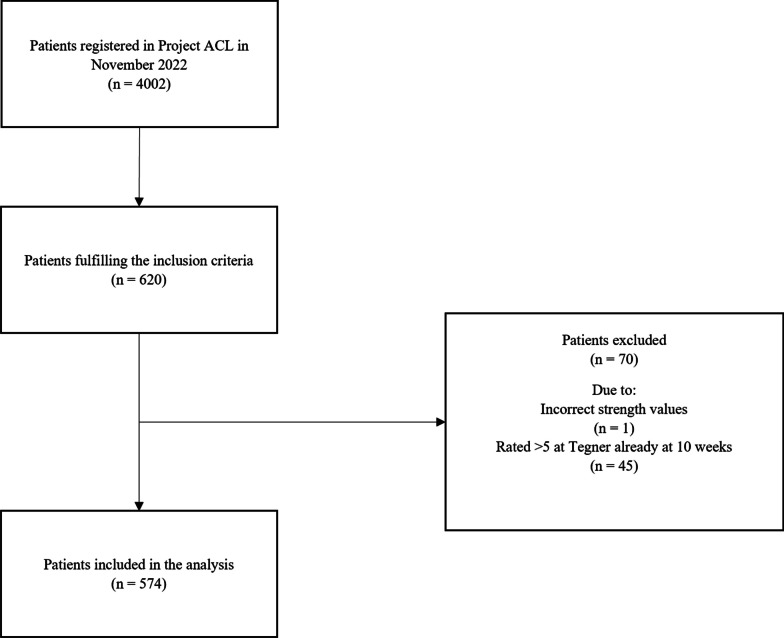
Flowchart of inclusion/exclusion. *Tegner* Tegner activity level scale, *n* numbers

A total of 574 patients (50.0% female) with a mean age of 24.0 ± 9.4 years at primary ACL reconstruction met the inclusion criteria (Fig. 1). The hamstring tendon autograft was most frequently used (82.7%) compared with the patellar tendon autograft (17.3%). Patients returned to preinjury sport level at a mean of 12.8 ± 5.9 months (95% CI 12.3; 13.3 months) after ACL reconstruction (Table 1).

**Table 1:** Patient demographics and the hamstrings-to-quadriceps ratio in patients who returned to preinjury sport level

	Total (n = 574)	Re-injury within 2 years from RTS (n = 64)	No re-injury within 2 years (n = 510)	Difference between groups Mean (95% CI)	*p* value
Sex, n (%)					
Female	287 (50.0%)	26 (40.6%)	261 (51.2%)	− 10.6 (− 24.2; 3.1)	0.14
Age at primary reconstruction (years)	24.0 (9.4)	19.8 (4.7)	24.6 (9.7)	− 4.8 (− 7.2; − 2.4)	**0.002**
Body Mass Index, (kg/m^2^)	23.6 (3.1)	23.8 (5.0)	23.6 (2.8)	0.2 (− 0.7; 1.0)	0.51
Graft choice, n (%)					
Hamstring tendon autograft	463 (82.7%)	56 (90.3%)	407 (81.7%)	8.6 (− 0.4; 17.6)	0.12
Patellar tendon autograft	97 (17.3%)	6 (9.7%)	91 (18.3%)		
Time to return to preinjury sport level (months)	12.8 (5.9) (12.2; 13.3)	11.7 (4.7) (10.6; 12.9)	12.9 (6.0) (12.4; 13.5)	− 1.2 (− 2.8; 0.3)	0.13
Side of second ACL injury (%)					
Ipsilateral		36 (56.3%)			
Contralateral		28 (43.8%)			
HQ-ratio (ACL reconstructed side) (%)	58.5 (12.3) (57.5; 59.5)	55.2 (10.6) (52.6; 57.9)	58.9 (12.4) (57.8; 60.0)	− 3.65 (− 6.78; − 0.44)	**0.026**
HQ-ratio (uninjured side) (%)	54.7 (7.8) (54.1; 55.4)	53.5 (8.9) (51.2; 55.7)	54.9 (7.6) (54.2; 55.6)	− 1.44 (− 3.51; 0.47)	0.16
Difference in HQ-ratio between the ACL reconstructed and uninjured leg (%)	3.73 (11.34) (2.80; 4.66)	1.78 (10.56) (− 0.86; 4.42)	3.98 (11.43) (2.98; 4.97)	− 2.20 (− 5.31; 0.76)	0.14
Relative peak torque of hamstrings strength, reconstructed (N m kg^−1^)	1.52 (0.31) (1.49; 1.54)	1.46 (0.33) (1.38; 1.54)	1.52 (0.31) (1.50; 1.55)	− 0.06 (− 0.14; 0.02)	0.14
Relative peak torque of quadriceps strength, reconstructed (N m kg^−1^)	2.64 (0.50) (2.60; 2.68)	2.68 (0.54) (2.55; 2.81)	2.63 (0.50) (2.59; 2.68)	− 0.05 (− 0.09; 0.20)	0.49
Hamstrings LSI (%)	98.0 (12.1) (97.0; 99.0)	97.7 (12.4) (94.5; 100.8)	98.0 (12.1) (96.9; 99.0)	− 0.35 (− 3.80; 2.79)	0.86
Quadriceps LSI (%)	92.9 (12.5) (91.8; 93.9)	95.0 (12.8) (91.8; 98.2)	92.6 (12.5) (91.5; 93.7)	2.44 (− 0.59; 5.79)	0.13

A bold p-value indicates a significance (*p* < 0.05). For categorical variables, n (%) is presented. For continuous variables, the mean (SD)/ (95% CI for mean) is presented

*ACL* anterior cruciate ligament, *CI* confidence intervals, *HQ* hamstrings-to-quadriceps, *kg* kilograms, *LSI* limb symmetry index, *M* meters, *n* numbers, *N m* Newton meters

### Second ACL Injury Upon Returning to Preinjury Sport Level

There were 64 (11.1%) second ACL injuries during the first 2 years after return to preinjury sport level. Of these 64 s ACL injuries, 36 (56.3%) were ipsilateral and 28 (43.8%) contralateral. There was a significantly lower HQ-ratio in the ACL reconstructed leg of patients who sustained a second ACL injury compared with those that did not (− 3.6%, 95% CI − 6.8; − 0.4%), at the time of return to preinjury sport level (Table 1).

### Odds for Sustaining a Second ACL Injury

For every 1% increase in the HQ-ratio of the ACL reconstructed leg, the odds of sustaining a second ACL injury, regardless of ipsi- or contralateral ACL injury, decreased by 3% (OR 0.97 [95% CI 0.95–1.00], *p* = 0.025). When adjusting for prognostic factors of a second ACL injury, the results were no longer significant (OR 0.98 [95% CI 0.95–1.01], *p* = 0.16). Furthermore, the area under the ROC-curve was 0.60 (0.52–0.67), suggesting a poor discriminatory ability of the HQ-ratio to identify those who would sustain a second ACL injury (Table 2). Fourteen patients (2.4%) had missing data for graft choice, and one patient (0.002%) had missing data for BMI.

**Table 2:** Hamstrings-to-quadriceps strength ratio for the odds of sustaining a second ACL injury

	Value	n (%) of event	Univariable*		Area under ROC curve (95%CI)	Multivariable**	
			OR (95%CI)	*p* value		OR (95%CI)	*p* value
HQ-ratio (ACL reconstructed side) (%)	30.9–< 52.8	33 (17.2%)					
	52.8–< 61.0	14 (7.3%)					
	61.0–139.0	17 (8.9%)	0.97 (0.95–1.00)	**0.025**	0.60 (0.52–0.67)	0.98 (0.95–1.01)	0.16
Time to RT preinjury sport level (months)	3.5–< 11.4	25 (13.1%)					
	11.4–< 12.8	23 (12.0%)					
	12.8–24.7	16 (8.4%)	0.96 (0.92–1.01)	0.13	0.54 (0.47–0.61)	0.96 (0.91–1.01)	0.12
Sex, n (%)	Male	38 (13.2%)					
	Female	26 (9.1%)	0.65 (0.38–1.11)	0.11	0.55 (0.49–0.62)	0.73 (0.41–1.30)	0.28
Graft choice, n (%)	Hamstring tendon autograft	56 (12.1%)					
	Patellar tendon autograft	6 (6.2%)	0.48 (0.20–1.15)	0.098	0.54 (0.50–0.58)	0.52 (0.20–1.34)	0.18
Age at primary reconstruction, n (%)	12.9–< 18.3	31 (16.2%)					
	18.3–< 24.9	23 (12.0%)					
	24.9–68.0	10 (5.2%)	0.92 (0.87–0.96)	**0.0002**	0.64 (0.58–0.71)	0.94 (0.89–0.98)	**0.008**
Tegner preinjury, n (%)	6–< 8	9 (5.1%)					
	8–< 9	23 (14.8%)					
	9–10	32 (13.1%)	1.32 (1.06–1.66)	**0.015**	0.59 (0.52–0.65)	1.17 (0.89–1.52)	0.26
Body mass index, n (%)	15.8–< 22.5	25 (13.1%)					
	22.5–< 24.5	18 (9.3%)					
	24.5–59.2	21 (11.1%)	1.02 (0.94–1.10)	0.60	0.48 (0.40–0.55)		

A bold p-value indicates a significance (*p* < 0.05). *ACL* anterior cruciate ligament, *CI* confidence intervals, *HQ* hamstrings-to-quadriceps, *n* numbers, *OR* odds ratio, *ROC* receiver operating curve, *RT* return to, *Tegner* tegner activity scale

*All analyses were performed with univariable logistic regression

**Multivariable logistic regression model including the hamstrings-to-quadriceps strength ratio (ACL reconstructed side), time to return to preinjury sport level (months), sex, graft choice, age at index operation and Tegner preinjury. Area under ROC curve with 95% CI for multivariable model = 0.69 (0.62–0.76)

### Ipsilateral and Contralateral ACL Injuries

The HQ-ratio on the ACL reconstructed side was not a significant predictor for sustaining an ipsilateral ACL injury, nor was the HQ-ratio on the uninjured side a predictor for sustaining a contralateral ACL injury (Table 3).

**Table 3:** Hamstrings-to-quadriceps strength ratio for the odds of sustaining an ipsilateral and contralateral anterior cruciate ligament injury

	Value	n (%) of event	Univariable*		Area under ROC curve (95%CI)	Multivariable**	
			OR (95%CI)	*p* value		OR (95%CI)	*p* value
*Ipsilateral ACL injuries*							
HQ-ratio (ACL reconstructed side) (%)	30.9–< 52.9	19 (10.4%)					
	52.9–< 61.1	7 (3.8%)					
	61.1–139.0	10 (5.5%)	0.97 (0.94–1.00)	0.051	0.60 (0.51–0.70)	0.97 (0.94–1.01)	0.12
Tegner preinjury n (%)	6–< 8	6 (3.5%)					
	8–< 9	13 (5.5%)					
	9–10	17 (7.4%)	1.28 (0.96–1.71)	0.097	0.58 (0.49–0.66)	1.10 (0.79–1.53)	0.58
*Contralateral ACL injuries*							
HQ-ratio (uninjured side) (%)	32.0–< 51.6	14 (7.8%)					
	51.6–< 57.7	6 (3.3%)					
	57.7–104.3	8 (4.5%)	0.97 (0.92–1.02)	0.19	0.58 (0.45–0.70)	0.97 (0.92–1.02)	0.24
Tegner preinjury n (%)	6–< 8	3 (1.8%)					
	8–< 9	10 (7.0%)					
	9–10	15 (6.6%)	1.37 (0.98–1.92)	0.063	0.60 (0.52–0.68)	1.39 (0.99–1.95)	0.056

A bold p-value indicates a significance (*p* < 0.05). *ACL* anterior cruciate ligament, *CI* confidence intervals, *HQ* hamstrings-to-quadriceps, *n* numbers, *OR* odds ratio, *ROC* receiver operating curve, *Tegner* Tegner activity scale

*All analyses were performed with univariable logistic regression

**Multivariable logistic regression model including hamstrings-to-quadriceps strength ratio (ACL reconstructed side) and Tegner preinjury. Area under the ROC curve with 95% CI for multivariable model = 0.66 (0.57–0.74)

### Relative Peak Torque and Limb Symmetry Index

The relative peak torque of hamstrings and quadriceps strength on the ACL reconstructed leg was not significantly associated with the odds of a second ACL injury. Furthermore, neither hamstrings nor quadriceps LSI were significantly associated with the odds of a second ACL injury (Table 4). Four patients (0.007%) had missing data for relative peak torque of hamstring and quadriceps strength on the reconstructed side.

**Table 4:** Post-hoc logistic regression analysis of association between relative peak torque of the hamstrings and quadriceps strength on the ACL reconstructed side, and the LSI for the hamstrings and quadriceps with second ACL injuries

	Value	n (%) of event	Univariable*		Area under ROC curve (95%CI)	Multivariable**	
			OR (95%CI)	*p* value		OR (95%CI)	*p* value
HQ-ratio (ACL reconstructed side) (%)	30.9–< 52.8	33 (17.2%)					
	52.8–< 61.0	14 (7.3%)					
	61.0–139.0	17 (8.9%)	0.97 (0.95–1.00)	**0.025**	0.60 (0.52–0.67)	0.98 (0.95–1.01)	0.16
Relative peak torque of hamstrings strength, reconstructed (N m kg^−1^)	0.33–< 1.37	29 (15.3%)					
	1.37–< 1.64	16 (8.4%)					
	1.64–2.51	19 (10.0%)	0.52 (0.22–1.21)	0.13	0.57 (0.49–0.64)	0.47 (0.18–1.27)	0.14
Relative peak torque of quadriceps strength, reconstructed (N m kg^−1^)	1.06–< 2.42	19 (10.0%)					
	2.42<–2.85	19 (10.0%)					
	2.85–4.07	26 (13.7%)	1.21 (0.72–2.03)	0.47	0.53 (0.45–0.61)	0.96 (0.52–1.78)	0.91
LSI hamstrings (%)	42.5–< 93.0	19 (9.9%)					
	93.0–< 102.5	25 (13.1%)					
	102.5–140.0	20 (10.4%)	1.00 (0.98–1.02)	0.83	0.52 (0.44–0.59)	1.00 (0.98–1.02)	0.91
LSI quadriceps (%)	40.2–< 90.2	22 (11.5%)					
	90.2–< 98.7	15 (7.8%)					
	98.7–129.4	27 (14.1%)	1.02 (0.99–1.04)	0.14	0.55 (0.47–0.63)	1.01 (0.98–1.03)	0.68

A bold p-value indicates a significance (*p* < 0.05). *ACL* anterior cruciate ligament, *CI* confidence intervals, *HQ* hamstrings-to-quadriceps, *LSI* limb symmetry index, *n* numbers, *N m* Newton meters, *OR* odds ratio, *ROC* receiver operating curve

*All analyses were performed with univariable logistic regression

**Multivariable logistic regression model including hamstrings-to-quadriceps strength ratio (ACL-reconstructed side), relative peak torque of hamstrings strength, relative peak torque of quadriceps strength, LSI hamstrings, and LSI quadriceps. Area under ROC curve with 95% CI for multivariable model = 0.69 (0.62–0.76)

### Sensitivity Analysis

There was a 27% increase in the odds of a second ACL injury for every 10% decrease in the HQ-ratio, (OR 0.73 (0.56; 0.94), *p* = 0.015). No significant results were found when adjusting for prognostic factors (OR 0.81 (0.60; 1.09), *p* = 0.17). The area under the ROC-curve was 0.51 (0.53; 0.68), indicating poor discriminatory ability.

### Difference in the Hamstrings-to-Quadriceps Strength Ratio Between Sexes

Females had a significantly higher HQ-ratio compared with males in both the ACL reconstructed (60.3% ± 13.7% vs. 56.6% ± 10.3%, *p* = 0.0002) and uninjured leg (56.4% ± 8.0% vs. 53.1% ± 7.1%, *p* < 0.001) at the time of return to preinjury sport level (Table 5).

**Table 5:** Differences in hamstrings-to-quadriceps strength ratio between sexes

	Total (n = 574)	Male (n = 287)	Female (n = 287)	*p* value	Difference between groups Mean (95% CI)
HQ-ratio (ACL reconstructed side) (%)	58.5 (12.3) (57.5; 59.5)	56.6 (10.3) (55.4; 57.8)	60.3 (13.7) (58.7; 61.9)	**0.0002**	− 3.74 (− 5.71; − 1.76)
HQ-ratio (uninjured side) (%)	54.7 (7.8) (54.1; 55.4)	53.1 (7.1) (52.2; 53.9)	56.4 (8.0) (55.5; 57.3)	**< 0.001**	− 3.34 (− 4.60; − 2.10)
Difference in HQ-ratio between the ACL reconstructed and uninjured leg (%)	3.73 (11.34) (2.80; 4.66)	3.41 (9.57) (2.22; 4.60)	3.87 (13.16) (2.26; 5.49)	0.68	− 0.40 (− 2.25; 1.48)

A bold p-value indicates a significance (*p* < 0.05). For continuous variables, the mean (SD)/(95% CI) is presented

*ACL* anterior cruciate ligament, *CI* confidence intervals, *HQ* hamstrings-to-quadriceps, *n* numbers

## Discussion

The main findings in this study were that (1) the HQ-ratio did not affect the odds for sustaining a second ACL injury after return to preinjury sports level, and (2) females had a higher HQ-ratio compared with males for both the ACL reconstructed and uninjured side.

### Hamstrings-to-Quadriceps Strength Ratio and the Odds of a Second ACL Injury

Despite the development of test batteries to provide patients and caregivers with information regarding the patient’s recovery of muscle strength and function prior to RTS, the occurrence of a second ACL injury after returning to pivoting sports is alarmingly high, with an injury rate ranging from 18 to 42% at a follow-up time of 10 years [18, 19]. Historically, the quadriceps and hamstrings strength have been evaluated as the LSI and in isolation [1], with little regard to the HQ-ratio. In our results, we found that for every 1% increase in the HQ-ratio, the odds of a second ACL injury decreased by 3%. However, sustaining a second ACL injury is naturally not due to a single factor but the result of multiple interacting factors. In the multivariable analysis, the result was no longer significant. Thus, the HQ-ratio did not significantly affect the odds for a second ACL injury upon returning to preinjury sports level. This is not surprising, given the many theories and risk factors that have been proposed to explain the mechanism of non-contact ACL injury in addition to the HQ-ratio, including (but not limited to) excessive knee valgus, abduction moments, axial compressive forces on the knee-joint and neurocognitive errors [20–22]. Nevertheless, the assumption that the HQ-ratio would influence the odds for a second ACL injury is founded on the theoretically synergistic role of the hamstrings to resist anterior tibial translation and thus decrease the load on the ACL. In other words, to rely solely on the HQ-ratio of the ACL reconstructed side to estimate the odds of sustaining a second ACL injury, regardless of ipsi-or contralateral ACL injury, may be misleading. Consequently, for every 1% increase in the HQ-ratio of the ACL reconstructed side, the odds of an ipsilateral ACL injury decreased by 3% (95% CI 0.97 (0.94; 1.00), *p* = 0.051). However, after addressing prognostic factors in the multivariable analysis, the association between HQ-ratio and ipsilateral ACL injuries was no longer significant. The finding in the present study that the HQ-ratio did not affect the odds for a second ACL injury is in contrast with previous research [6, 23], and questions the use of the HQ-ratio as part of the decision-making prior to RTS.

In our cohort of 574 patients, 82.7% were reconstructed with hamstring tendon autografts. It is well established that harvesting hamstring tendons for ACL reconstruction leads to reduced knee flexor strength, while the patellar-and-quadriceps tendon autograft procedures lead to reduced knee extensor strength up to 1 year after reconstruction [24, 25]. In addition, patients with a hamstring tendon autograft have been reported to recover quadriceps strength sooner than patellar tendon autografts and allografts [26]. In our cohort, the patients who sustained a second ACL injury had a significantly lower HQ-ratio compared to those who did not sustain a second ACL injury (− 3.6%, 95% CI − 6.8; − 0.4%) which may reflect the predominance of patients treated with hamstring tendon autografts in our cohort. Nevertheless, the HQ-ratio did not significantly affect the odds of a second ACL injury. A lower HQ-ratio may be considered to be the tip of an iceberg; we may see it in patients who will sustain a second ACL injury, although factors such time to RTS, age, higher posterolateral tibial slope, greater knee laxity, and activity level/exposure may be of greater importance than the HQ-ratio [16].

### Relative Peak Torque and Differences Between Sexes

To only evaluate the ratio between the hamstrings and quadriceps provides insufficient information regarding (1) whether the patient has recovered muscle strength compared with the uninjured side and (2) whether the strength is sufficient in the hamstrings and the quadriceps, respectively. To address the relative peak torque, as well as whether patients had recovered their muscle strength with their uninjured limb as a reference (LSI), we performed a post-hoc logistic regression. However, we observed no additional significant odds ratios in our results.

In the analysis between females and males, we observed that females had a higher HQ-ratio compared with males in both the ACL reconstructed and uninjured side. This is in line with the findings of Myer et al. [7] who reported that females had a lower quadriceps strength than males, leading to a higher HQ-ratio. Despite differences in the HQ-ratio between sexes, no difference in the injury rate between sexes was observed in our cohort, highlighting the multifactorial nature of ACL injuries.

### Limitations

In the present study, we included patients who obtained the same score or higher on Tegner as preinjury, indicating a return to preinjury sports level. However, we do not know the kind of sport to which they returned. The HQ-ratio may differ between sports [27], as different sports impose different demands on the knee, e.g., primarily vertical forces such as in jumping, or more horizontal forces such as in sprinting [27]. Different sports may also contribute to limb dominance, e.g., being right footed in soccer, which may have affected our analysis between the legs. Although the patients included may have participated in different sports, we observed no significant difference in the HQ-ratio between the ACL reconstructed and uninjured leg, which is in line with previous research [28]. Furthermore, the strength tests of the quadriceps and hamstrings in Project ACL are performed with isokinetic concentric contractions in a seated position, at an angular velocity of 90°/s. The relevance of open-chain concentric strength tests to ACL injury situations can be questioned, as the hamstrings work in a closed chain during typical ACL injury situations, such as landing and side cutting maneuvers [21]. In these situations, the hamstrings may work eccentrically and/or isometrically to reduce the strain on the ACL. In addition to the contraction mode, the hip and knee angles, as well as the contraction velocity, will also differ in sporting situations. Previous research has reported that a more extended hip and deeper angle of knee flexion will produce a lower HQ-ratio compared with a flexed hip and lower angle of knee flexion [29]. Taken together, multiple aspects affect the HQ-ratio, making it complex to assess and interpret. It is possible that our way of determining the HQ-ratio in the present study may not be representative of the way the muscles work to reduce the strain on the ACL and this could have affected our analysis of the odds of a second ACL injury.

## Conclusion

Patients who sustain a second ACL injury within 2 years after return to preinjury sport level have a significantly lower HQ-ratio. However, the HQ-ratio did not significantly influence the odds for sustaining a second ACL injury after return to preinjury sport level. Consequently, the HQ-ratio should not be considered in isolation but in combination with other factors in RTS decision-making. Moreover, females had significantly higher HQ-ratio than males for both the ACL reconstructed and uninjured side.

## Data Availability

Data is available upon reasonable request.

## Abbreviations

ACL: Anterior cruciate ligament
CI: Confidence interval
HQ: Hamstrings-to-quadriceps
LSI: Limb symmetry index
OR: Odds ratio
RTS: Return to sport
ROC: Receiver operating characteristic
Tegner: Tegner activity scale
